# Inactivation Performance of Pseudorabies Virus as African Swine Fever Virus Surrogate by Four Commercialized Disinfectants

**DOI:** 10.3390/vaccines11030579

**Published:** 2023-03-02

**Authors:** Zheng Ni, Liu Chen, Tao Yun, Ronghui Xie, Weicheng Ye, Jionggang Hua, Yinchu Zhu, Cun Zhang

**Affiliations:** 1 State Key Laboratory for Managing Biotic and Chemical Threats to the Quality and Safety of Agro-Products, Institute of Animal Husbandry and Veterinary Sciences, Zhejiang Academy of Agricultural Sciences, Hangzhou 310021, China; 2 Zhejiang Provincial Center for Animal Disease Control, Hangzhou 310018, China

**Keywords:** evaluation model for disinfectant, inactivation performance, infection control, African swine fever virus (ASFV), pseudorabies virus (PRV)

## Abstract

This study was based on similar physicochemical characteristics of pseudorabies virus (PRV) and African swine fever virus (ASFV). A cellular model for evaluation of disinfectants was established with PRV as an alternative marker strain. In the present study, we evaluated the disinfection performance of commonly used commercialized disinfectants on PRV to provide a reference for the selection of good ASFV disinfectants. In addition, the disinfection (anti-virus) performances for four disinfectants were investigated based on the minimum effective concentration, onset time, action time, and operating temperature. Our results demonstrated that glutaraldehyde decamethylammonium bromide solution, peracetic acid solution, sodium dichloroisocyanurate, and povidone-iodine solution effectively inactivated PRV at concentrations 0.1, 0.5, 0.5, and 2.5 g/L on different time points 30, 5, 10, and 10 min, respectively. Specifically, peracetic acid exhibits optimized overall performance. Glutaraldehyde decamethylammonium bromide is cost effective but requires a long action time and the disinfectant activity is severely affected by low temperatures. Furthermore, povidone-iodine rapidly inactivates the virus and is not affected by environmental temperature, but its application is limited by a poor dilution ratio such as for local disinfection of the skin. This study provides a reference for the selection of disinfectants for ASFV.

## 1. Introduction

African swine fever (ASF) is an acute, severe, and highly contagious hemorrhagic viral disease with high morbidity and mortality caused by the African swine fever virus (ASFV) [1]. It has a short onset time causing up to 100% mortality in acute infections [2,3]. ASF mainly existed in Africa and was rarely introduced to other continents until 2007, when it was reported in Georgia [2]. Later, it rapidly spread across the Caucasus and into the Russian Federation and reached the European Union (EU) in 2014 [4]. In August 2018, ASF appeared in Northeast China and then spread rapidly across all provinces and municipalities, causing huge economic losses to the pig industry [5,6,7]. In China, small-scale pig farms with weak biosecurity systems account for a large proportion, which may be one reason for the rapid spread of ASF in China [8]. Hence, ASF can be regarded as a catastrophic swine disease threatening animal health, the economy, and social life [2]. However, so far, there are no commercialized, safe, and effective drugs or vaccines against ASF [9,10]. Currently, the core part of ASF prevention and control in China is focused on the biosafety prevention and control system. Specifically, appropriate disinfectants are selected to inactivate the virus and block transmission during the breeding, transportation, slaughtering, processing, inspection, and quarantine processes. Accordingly, identifying sensitive, safe, and efficient disinfectants against ASFV with suitable environmental conditions, exposure time, and temperature range is greatly needed [11]. As already proven, ASFV is very resistant to a wide variety of conditions and is one of the most complex swine viruses known to date. It is resistant to a broad range of pH (3.9–11.5) but can be quickly inactivated by high temperature (70 min at 56 °C or 20 min at 60 °C) and disinfectants, such as sodium hydroxide, hypochlorite, phenol, and iodine compounds [10]. Due to its infectivity, lack of vaccinations, and responsibility for serious economic and production losses, the disease is listed as notifiable by the World Organization for Animal Health (WOAH). Screening for sensitive, safe, and efficient disinfectants against ASFV has become an urgent demand in the pig industry.

ASFV is a member of the family Asfarviridae and the genus Asfivirus. It is a large, enveloped DNA virus. Its virions have a specific icosahedral structure of a protein capsid, which surrounds an internal membrane and a nucleoprotein core. It shows high similarity to other enveloped viruses in terms of particle size, genome size, and physicochemical properties [12,13,14]. The World Organization for Animal Health (WOAH) advice is based on the disinfection study results of other enveloped viruses and indicates that ASFV disinfectants must be chosen based on adequate outcomes [15,16,17]. Control measures nowadays include culling infected pigs and maintaining high biosecurity standards, which rely primarily on disinfection. The fundamental and most important aspect of biosecurity is disinfection and the proper use of disinfectants [18,19]. Inactivating pathogenic bacteria on objects or surfaces is one of the most effective techniques to prevent the spread of infectious diseases. The ideal disinfectant has the following qualities: quick action, durability, non-toxicity, and minimum environmental contamination risk [20]. Therefore, it is logistically more difficult to undertake such research when rapid dissemination of information is required. To avoid, respond to, and recover from infectious disease outbreaks, it is essential to disinfect surfaces contaminated with dangerous microorganisms.

In this context, the availability of data regarding the virucidal activity of chemical compounds against ASFV provides useful information to properly plan decontamination procedures aimed at preventing the introduction of ASFV and limiting its secondary spread [21]. The ideal disinfectant should have the advantages of fast and high efficiency, low toxicity, broad antimicrobial spectrum, and stable nature [22]. In this study, four commonly used disinfectants (glutaraldehyde decamethylammonium bromide, povidone-iodine solution, peracetic acid solution, and sodium dichloroisocyanurate) were selected based on different disinfection mechanisms. The “Technical Specifications for Disinfection” guidelines were followed to study the inactivation of PRV and the performances of the four disinfectants were evaluated. This study provides a reference for the selection of appropriate disinfectants in large-scale pig farms and the development of an ASF prevention and control disinfection system.

## 2. Materials and Methods

### 2.1. Cells and Viruses

The fluorescent-tag virus rPRV-ZJ was constructed and stored by our laboratory at the Zhejiang Academy of Agricultural Sciences (ZAAS) [23]. BHK-21 cells were stored in our laboratory, and were maintained in Dulbecco’s minimum essential medium (DMEM) (Corning Inc., Corning, NY, USA) supplemented with 10% fetal bovine serum (FBS) (Corning Inc.). The cultures were grown at 37 °C, in a humidified atmosphere of air containing 5% CO_2_.

### 2.2. Reagents

#### 2.2.1. Selection of Disinfectants

Four disinfectants, including commercially available quaternary ammonium salts, iodine preparations, chloride preparations, and peroxides, were selected in this study. All products were approved by the authority and did not expire during the study.

Glutaraldehyde decamethylammonium bromide solution (5 g of glutaraldehyde + 5 g of methyl ammonium per 100 mL, approval no. 230036245): the recommended dilutions were 1:2000–1:4000, 1:500–1:1000, and 1:1500–1:3000 for conventional environmental spraying disinfection, environmental spraying disinfection during the epidemic, and immersion disinfection of instruments and equipment, respectively.

Povidone-iodine solution (5% of iodine, g/mL, approval no. (2016) 240041575): the recommended concentrations were 5, 0.5–1.0, and 0.1% for skin disinfection and treatment of skin diseases, immersion disinfection of cow teats, and flushing of mucosa and wound, respectively.

Peracetic acid solution (20%, g/mL, approval no. 180206432: the recommended dilutions were 1:200–1:400 and 1:500 for spray disinfection of animal houses and immersion disinfection of food utensils of livestock and staff clothes, respectively; the recommended volume for spray disinfection of animal houses was 5~15 mL/m^2^.

Sodium dichloroisocyanurate powder (effective chloride = 20.0%, approval no. 180086051): the recommended concentrations were 2.0, 0.5, and 1.0 g/L for spray disinfection of animal houses and instrument, immersion disinfection of food utensils of livestock and staff clothes, and disinfection of epidemic focus, respectively.

#### 2.2.2. Neutralizers Corresponding to Different Disinfectants

The neutralizers corresponding to glutaraldehyde decamethylammonium bromide solution, povidone-iodine solution, peracetic acid solution and sodium dichloroisocyanurate were 0.1% Tween 80 + 0.3% glycine (in PBS), 0.1 mol/L sodium thiosulfate (in PBS), 0.1 mol/L sodium thiosulfate (in PBS) and 0.2 mol/L sodium thiosulfate (in PBS), respectively [20,24,25].

#### 2.2.3. Preparation of Standard Hard Water

All disinfectants and neutralizers used in this study were prepared using standard hard water following the Technical Specifications for Disinfection. Specifically, the standard hard water was prepared by adding 0.034 g of CaCl2 and 0.139 g of MgCl_2_•6H_2_O into 1000 mL of distilled water.

### 2.3. Proliferation and Measurement Titer of rPRV-ZJ on BHK-21 Cells

The rPRV-ZJ strain was cultured on BHK-21 cells for proliferation. After three freeze-thaw cycles, sample was centrifuged at 8000× *g* for 10 min at 4 °C and the virus supernatant was collected. The virus suspension was aliquoted and stored at −80 °C. The virus suspension samples were diluted 10 times (gradient dilution) using serum-free DMEM cell culture solution and inoculated on a 96-well culture plate covered by a single layer of BHK-21 cells. Each gradient had eight replicates containing 100 µL sample suspension. For negative control, samples were diluted with DMEM cell culture solution containing 2% FBS and cultured at 37 °C and 5% CO_2_ for 96 h consecutively; cytopathic effect (CPE) changes (if any) of cells were recorded. Additionally, TCID50/100 μL titration of the virus was calculated using the Reed–Muench method.

### 2.4. Effects of Neutralizer, Disinfectant, and Neutralized Products on the Growth of BHK-21 Cells

According to the Technical Specifications for Disinfection by the Ministry of Health, the People’s Republic of China, the effects of neutralizer, disinfectant, and neutralized products on the growth of BHK-21 cells were evaluated. Herein, four groups were established, including the disinfectant group (Group A), the neutralizer group (Group B), the neutralized products group (Group C), and the negative control group (Group D). All reagents were inoculated on BHK-21 cells, mixed, and then cultured for 3–4 h. Cell growth was observed under a microscope [26].

### 2.5. Establishment of Evaluation Model for Cell Disinfection

#### 2.5.1. Virus Disinfection

According to Technical Specifications for Disinfection 2002, a disinfectant solution was prepared of doubled concentration (2X), and then virus suspension of equal volume was added with mixing. Next, after the designated period of virus inactivation, a neutralizer solution of equal volume was added. After 10 min, the virus titer was measured to determine TCID50/100 μL. Herein, TCID50/100 μL of the original virus suspension and the sample solution is denoted by N0 and Nx, respectively.

Virus inactivation rate = (log N0 − log Nx)/log N0 × 100%

#### 2.5.2. Measurements of Minimum Effective Content of Disinfectant

The disinfectant solution was mixed with the original virus suspension in equal volumes and kept at room temperature for 30 min. Then, a neutralizer solution of equal volume was added with mixing. After 10 min, TCID50 of the sample solution was measured and compared with that of the original virus suspension. The virus inactivation rate was calculated. Additionally, the disinfectant solution was diluted into the solutions of gradient concentrations and subjected to measurements as described above. The minimum effective concentration of the disinfectant is defined as the minimum disinfectant concentration corresponding to a 100% virus inactivation rate.

#### 2.5.3. Disinfection Performances Based on Onset and Action Time

We used the minimum effective concentration as the working concentration. Diluted disinfectant solutions were mixed with the original virus suspension in equal volumes and the mixtures were allowed to stand for 1, 5, 10, and 30 min, respectively. Next, an equal volume of neutralizer solution was added. After 10 min, the virus inactivation rates were measured.

#### 2.5.4. Effects of Temperature on Disinfection Performances

The measured minimum effective concentrations were used as working concentrations. The sample solutions were kept untouched at 4 and 37 °C for 30, 60, and 180 min, respectively. Next, an equal volume of neutralizer solution was added and the virus inactivation rates in BHK-21 cells were measured after 10 min.

## 3. Results

### 3.1. Proliferation of rPRV-ZJ in BHK-21 Cells and Measurement of TCID50

**Figure 1:**
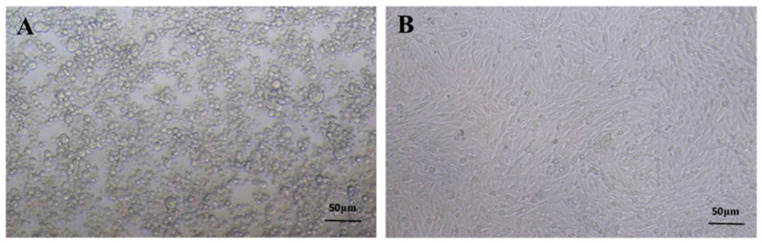
The proliferation of PRV in BHK-21 cells. (**A**) PRV-infected BHK-21 cells. (**B**) Normal BHK-21 cells.

The frozen rPRV-ZJ strain was sub-cultured in BHK-21 cells resulting in severe cytopathy (larger, round, and vacuolar cells) after 24 h (Figure 1A,B). The virus titer was calculated using the Reed–Muench method [27]. TCID50 of rPRV-ZJ was 10^7.25^/100 μL.

### 3.2. Effects of Neutralizers, Disinfectants, and Neutralized Products on the Growth of BHK-21 Cells

BHK-21 cells inoculated with different disinfectants, neutralizers, and neutralized products were characterized by an inverted microscope. Compared with the negative control group, inoculated BHK-21 cells exhibited regular morphology and good growth. This demonstrated that disinfectants, neutralizers, and neutralized products have negligible effects on cell growth suggesting no cellular toxicity.

### 3.3. Effects of Disinfectant on Virus Proliferation

**Figure 2:**
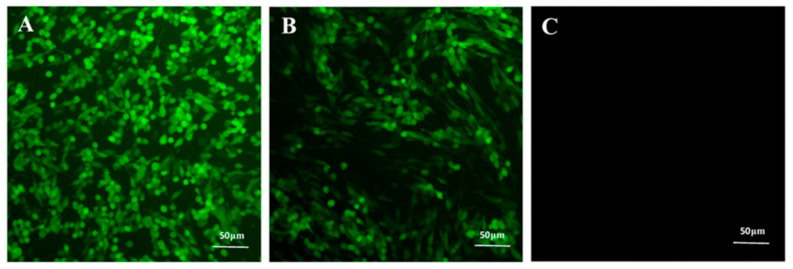
Effects of disinfectants on virus proliferation. (**A**) Fluorescence of BHK-21 cells inoculated with PRV. (**B**) PRV and disinfectant after 24 h. (**C**) Fluorescence of BHK-21 cells after complete virus inactivation.

Following 24 h after virus inoculations, over 90% of the BHK-21 cells exhibited green fluorescence. However, the BHK-21 cells inoculated with both PRV and disinfectants showed significantly lower or negligible green fluorescence, indicating complete inactivation of the virus (Figure 2). The results demonstrate that disinfectants inactivated the virus.

### 3.4. Minimum Effective Content of Four Disinfectants

**Figure 3:**
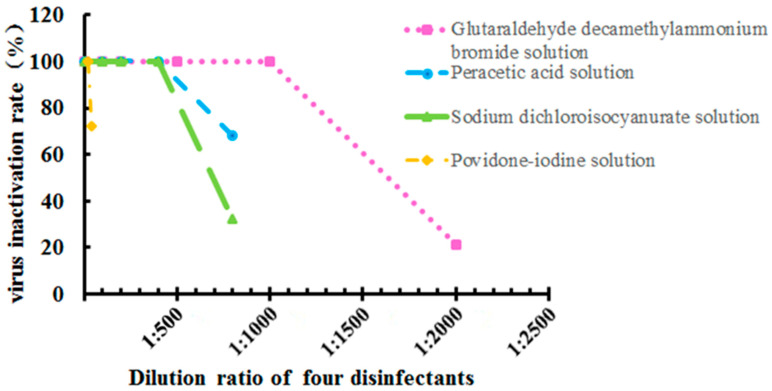
Virus inactivation rate for four disinfectants at different dilutions and determination of the minimum effective content. Data were presented as mean ± SD.

Next, virus inactivation rates for the four disinfectants at different dilutions and their respective active components were investigated for 100% virus inactivation rate at room temperature for 30 min. The best dilution and effective content of glutaraldehyde decamethylammonium bromide solution were 1:1000 dilution and 0.1 g/L; the best dilution and effective contents of peracetic acid solution and sodium dichloroisocyanurate were 1:400 dilutions and 0.5 g/L, and the best dilution and effective content of the povidone-iodine solution was 1:20 dilution and 2.5 g/L, respectively. Figure 3 illustrates the virus inactivation rates for the four disinfectants at different dilution rates.

### 3.5. Disinfection Performances Based on Onset and Action Time

**Figure 4:**
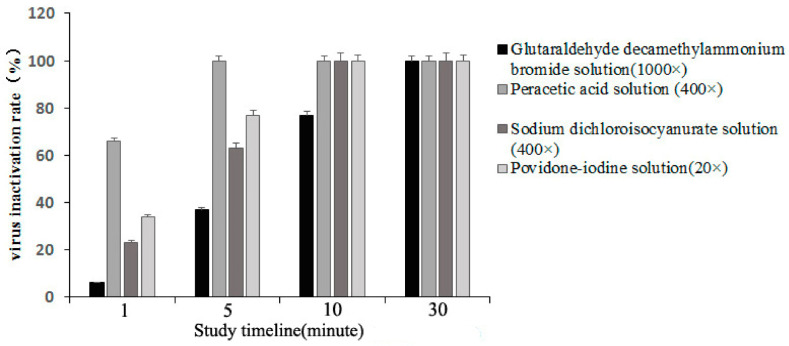
The shortest onset time of four disinfectants and the influence of different action times on the disinfection effect at room temperature.

The peracetic acid solution with an effective content of 0.5 g/L had a virus inactivation rate of 66 and 100% at room temperature treatment for 1 and 5 min, respectively. Likewise, sodium dichloroisocyanurate (0.5 g/L) and povidone-iodine solution (2.5 g/L) had a virus inactivation rate of 100% at room temperature treatment for 10 min. The glutaraldehyde decamethylammonium bromide solution (0.1 g/L) with 1000 times dilution had PRV inactivation rates of 34, 77, and 100% after 5, 10, and 30 min treatments at room temperature, respectively (Figure 4).

### 3.6. Effects of Temperature on Disinfection Performances of Disinfectants

The results indicated that the disinfection performance of glutaraldehyde decamethylammonium bromide solution degraded drastically at low temperatures (Table 1). After 30 and 60 min of treatments, the PRV inactivation rates were 27% at 4 °C; after 180 min, the virus inactivation rate was only 70%. The disinfection performance of the peracetic acid, sodium dichloroisocyanurate, and povidone-iodine was independent of temperature and the virus inactivation rate reached 100% after 30 min of treatment.

## 4. Discussion

ASFV has possibly become the most relevant epizootic disease from an animal health perspective. Swine viral infections are acute and spread rapidly and severely, affecting the breeding industry and agricultural economy. Meanwhile, these diseases are also severe hazards to public health safety. Besides conventional vaccine immunization, the core prevention strategy against these viral infections remains the construction of an efficient biosafety system, such as the development of rational disinfection technology [28]. The highest level of chemical substance efficiency depends on proper use, the mechanism of action, and application parameters such as concentration, contact time, pH, temperature, and the presence of organic matter. Therefore correct disinfectant selection and application are essential [28].

Efficient, toxic-free, reliable, and cost-effective disinfectants may play a key role in blocking the spread of pathogenic microorganisms reducing the probability of animal infection [29]. Especially, the outbreak of ASF has brought a huge loss to the breeding industry. However, so far, no commercialized vaccines or effective treatment has been reported for ASF; only chemical disinfection can be used [30,31]. Due to similar physiochemical characteristics, PRV, which is also a lipid-enveloped virus, can be employed as an alternative marker strain to evaluate the disinfection performance of different disinfectants [31,32]. In this study, the four most commonly used disinfectants in pig farms were evaluated for disinfection performances based on relevant operating procedures of the WOAH reference laboratory. Cell disinfection evaluation models and systems were established. This study provides a reference for the rational disinfection and control of ASFV in China.

As a Cl-containing disinfectant, sodium dichloroisocyanurate was decomposed into hypochlorous acid and cyanuric acid in water. The structures of virus particles were destroyed by oxidation causing virus inactivation. The results indicated that sodium dichloroisocyanurate powder (1:400) exhibited good disinfection performance showing inactivation rates of 65% and 100% after 5 and 10 min treatments at room temperature, respectively. However, the disinfectant dilution was lower than that recommended in the instructions. This may be attributed to decomposition during storage suggesting that sodium dichloroisocyanurate powder performance decreases after long storage. Glutaraldehyde decamethylammonium bromide, a quaternary ammonium salt disinfectant, is a cationic surfactant that does not irritate animal skin and tissues. The study revealed that glutaraldehyde decamethylammonium bromide required low dosages. Specifically, the virus inactivation rate of glutaraldehyde decamethylammonium bromide (1: 1000) was 100% at room temperature treatment for 30 min. Hence, it is suitable for the disinfection of large sites. However, glutaraldehyde decamethylammonium bromide is highly sensitive to low temperatures. The virus inactivation rates decreased to 27 and 70% at 4 °C for 30 min and 3 h treatments, respectively. A 100% virus inactivation rate could be achieved only at 1:500 dilutions. Povidone-iodine, a loose complex of iodine and polyvinylpyrrolidone, has low toxicity which depolymerizes in the solvent to release iodine. This slow-release effect elicits a long-term disinfection effect [33,34]. Povidone-iodine can rapidly inactivate viruses at room temperature and its disinfection performance was consistent at both low and high temperatures. However, povidone-iodine is limited by lower dilutions. Specifically, a 20-time dilution is a maximum dilution to achieve good disinfection performance. At 1:40 dilution, the virus inactivation rate of povidone-iodine was 79% even after 120 min. Hence, povidone-iodine cannot be used for large sites. Peracetic acid, a disinfectant of broad spectrum, high efficiency, and low toxicity can inactivate viruses in an extremely short time by oxidation. Moreover, its disinfection performance is not affected by temperature. Lin et al. [35] revealed that peracetic acid can rapidly inactivate microorganisms on the inner wall of the simulated lumen only causing limited corrosion to metallic materials.

Compared with non-enveloped viruses, ASFV and PRV, which are both enveloped viruses, can be readily inactivated by chemical disinfectants [36,37]. Additionally, enveloped viruses have poor stability outside the host [38,39]. In this study, PRV was inactivated by all four disinfectants, however, the required concentrations and action times are different. Nonetheless, this study was conducted under ideal conditions. In practice, feces, bedding, straw, and feed can cause resistance to chemical disinfectants [40]. To increase the effectiveness of the measure, it is necessary to perform a cleaning step prior to proper disinfection in order to remove organic matter. Hence, disinfectants shall be applied after thorough mechanical cleaning.

## 5. Conclusions

Due to the similar physiochemical characteristics of PRV with ASFV, the performances of four commonly used disinfectants without organic substances were evaluated using cell culture-based techniques, and PRV as an alternative marker strain. The results demonstrated that all disinfectants used in this study, under the conditions stated, inactivated PRV; the recommended dosages (optimized effective concentration and action time) of glutaraldehyde decamethylammonium bromide solution (0.1 g/L, 30 min), peracetic acid solution (0.5 g/L, 5 min), sodium dichloroisocyanurate (0.5 g/L, 10 min), and povidone-iodine solution (2.5 g/L, 10 min) were estimated. Among them, peracetic acid showed better action time, onset time, and reliability at both high and low temperatures. In practical applications, the use of disinfectant mixtures that are both effective and safe can further boost the overall efficacy of the disinfection process. This study provides a practical and theoretical reference for the establishment of ASFV disinfection standards.

## Figures and Tables

**Table 1:** The effect of temperature on disinfection performances of the disinfectants.

Disinfectant Category	Effective Content (g/mL)	Virus Inactivation Rate (%)			
		Action Temperature	Action Time		
			30 min	60 min	180 min
Glutaraldehyde decamethylammonium bromide solution	1 × 10^−4^	4 °C	27	27	70
		37 °C	100	100	100
Peracetic acid solution	5 × 10^−4^	4 °C	100	100	100
		37 °C	100	100	100
Sodium dichloroisocyanurate solution	5 × 10^−4^	4 °C	100	100	100
		37 °C	100	100	100
Povidone-iodine solution	2.5 × 10^−3^	4 °C	100	100	100
		37 °C	100	100	100

## Data Availability

The data may be provided on reasonable request to the corresponding author.

## References

[1] Costard S., Mur L., Lubroth J., Sanchez-Vizcaino J.M., Pfeiffer D.U. (2013). Epidemiology of African Swine Fever Virus. Virus Res..

[2] Sánchez-Cordón P.J., Montoya M., Reis A.L., Dixon L.K. (2018). African Swine Fever: A Re-Emerging Viral Disease Threatening the Global Pig Industry. Vet. J..

[3] Mazur-Panasiuk N., Żmudzki J., Woźniakowski G. (2019). African Swine Fever Virus—Persistence in Different Environmental Conditions and the Possibility of Its Indirect Transmission. J. Vet. Res..

[4] Cwynar P., Stojkov J., Wlazlak K. (2019). African Swine Fever Status in Europe. Viruses.

[5] Zhou X., Li N., Luo Y., Liu Y., Miao F., Chen T., Zhang S., Cao P., Li X., Tian K. (2018). Emergence of African Swine Fever in China, 2018. Transbound. Emerg. Dis..

[6] Zhao D., Liu R., Zhang X., Li F., Wang J., Zhang J., Liu X., Wang L., Zhang J., Wu X. (2019). Replication and Virulence in Pigs of the First African Swine Fever Virus Isolated in China. Emerg. Microbes Infect..

[7] Ge S., Li J., Fan X., Liu F., Li L., Wang Q., Ren W., Bao J., Liu C., Wang H. (2018). Molecular Characterization of African Swine Fever Virus, China, 2018. Emerg. Infect. Dis..

[8] Eblé P.L., Hagenaars T.J., Weesendorp E., Quak S., Moonen-Leusen H.W., Loeffen W.L.A. (2019). Transmission of African Swine Fever Virus via Carrier (Survivor) Pigs Does Occur. Vet. Microbiol..

[9] Gallardo C., Sánchez E.G., Pérez-Núñez D., Nogal M., de León P., Carrascosa Á.L., Nieto R., Soler A., Arias M.L., Revilla Y. (2018). African Swine Fever Virus (ASFV) Protection Mediated by NH/P68 and NH/P68 Recombinant Live-Attenuated Viruses. Vaccine.

[10] Dixon L.K., Stahl K., Jori F., Vial L., Pfeiffer D.U. (2020). African Swine Fever Epidemiology and Control. Annu. Rev. Anim. Biosci..

[11] Kalenic S., Horvatic J., Lynch P. (2005). Networking Hospital Infection Control in 10 Countries in Southeast Europe. Am. J. Infect. Control.

[12] Arias M., de la Torre A., Dixon L., Gallardo C., Jori F., Laddomada A., Martins C., Parkhouse R.M., Revilla Y., Rodriguez F. (2017). Approaches and Perspectives for Development of African Swine Fever Virus Vaccines. Vaccines.

[13] Fiori M.S., Sanna D., Scarpa F., Floris M., Di Nardo A., Ferretti L., Loi F., Cappai S., Sechi A.M., Angioi P.P. (2021). A Deeper Insight into Evolutionary Patterns and Phylogenetic History of Asfv Epidemics in Sardinia (Italy) through Extensive Genomic Sequencing. Viruses.

[14] Schulz K., Schulz J., Staubach C., Blome S., Nurmoja I., Conraths F.J., Sauter-Louis C., Viltrop A. (2021). African Swine Fever Re-Emerging in Estonia: The Role of Seropositive Wild Boar from an Epidemiological Perspective. Viruses.

[15] Shirai J., Kanno T., Tsuchiya Y., Mitsubayashi S., Seki R. (2000). Effects of Chlorine, Iodine, and Quaternary Ammonium Compound Disinfectants on Several Exotic Disease Viruses. J. Vet. Med. Sci..

[16] Gallina L., Scagliarini A. (2010). Virucidal Efficacy of Common Disinfectants against Orf Virus. Vet. Rec..

[17] Jeffrey D.J. (1995). Chemicals Used as Disinfectants: Active Ingredients and Enhancing Additives. Rev. Sci. Tech..

[18] Dellanno C., Vega Q., Boesenberg D. (2009). The Antiviral Action of Common Household Disinfectants and Antiseptics against Murine Hepatitis Virus, a Potential Surrogate for SARS Coronavirus. Am. J. Infect. Control.

[19] Hulkower R.L., Casanova L.M., Rutala W.A., Weber D.J., Sobsey M.D. (2011). Inactivation of Surrogate Coronaviruses on Hard Surfaces by Health Care Germicides. Am. J. Infect. Control.

[20] Juszkiewicz M., Walczak M., Wozniakowski G. (2019). Characteristics of Selected Active Substances Used in Disinfectants and Their Virucidal Activity against ASFV. J. Vet. Res..

[21] Jiang C., Gang S.U.N., Zhang F., Ai X., Feng X.-N., Hu W., Zhang X.-F., Zhao D.-M., Bu Z.-G., He X.-J. (2021). Viricidal Activity of Several Disinfectants against African Swine Fever Virus. J. Integr. Agric..

[22] Bicknell D.L., Jain R.K. (2001). Ozone Disinfection of Drinking Water—Technology Transfer and Policy Issues. Environ. Eng. Policy.

[23] Wales A.D., Gosling R.J., Bare H.L., Davies R.H. (2021). Disinfectant Testing for Veterinary and Agricultural Applications: A Review. Zoonoses Public Health.

[24] Böttcher B., Sarg B., Lindner H.H., Nagl M. (2017). Inactivation of Microbicidal Active Halogen Compounds by Sodium Thiosulphate and Histidine/Methionine for Time-Kill Assays. J. Microbiol. Methods.

[25] Martel J.A., Chatterjee P., Coppin J.D., Williams M., Choi H., Stibich M., Simmons S., Passey D., Jinadatha C. (2021). Capturing Portable Medical Equipment Disinfection Data via an Automated Novel Disinfection Tracking System. Am. J. Infect. Control.

[26] Reed L.J., Muench H. (1938). A Simple Method of Estimating Fifty per Cent Endpoints. Am. J. Epidemiol..

[27] Bellini S., Rutili D., Guberti V. (2016). Preventive Measures Aimed at Minimizing the Risk of African Swine Fever Virus Spread in Pig Farming Systems. Acta Vet. Scand..

[28] Juszkiewicz M., Walczak M., Mazur-Panasiuk N., Woźniakowski G. (2020). Effectiveness of Chemical Compounds Used against African Swine Fever Virus in Commercial Available Disinfectants. Pathogens.

[29] De Lorenzi G., Borella L., Alborali G.L., Prodanov-Radulović J., Štukelj M., Bellini S. (2020). African Swine Fever: A Review of Cleaning and Disinfection Procedures in Commercial Pig Holdings. Res. Vet. Sci..

[30] Karger A., Pérez-Núñez D., Urquiza J., Hinojar P., Alonso C., Freitas F.B., Revilla Y., Le Potier M.F., Montoya M. (2019). An Update on African Swine Fever Virology. Viruses.

[31] Sánchez-Vizcaíno J.M., Mur L., Gomez-Villamandos J.C., Carrasco L. (2015). An Update on the Epidemiology and Pathology of African Swine Fever. J. Comp. Pathol..

[32] Black D.N., Brown F. (1976). Purification and Physicochemical Characteristics of African Swine Fever Virus. J. Gen. Virol..

[33] Durani P., Leaper D. (2008). Povidone-Iodine: Use in Hand Disinfection, Skin Preparation and Antiseptic Irrigation. Int. Wound J..

[34] Flynn J. (2003). Povidone-Iodine as a Topical Antiseptic for Treating and Preventing Wound Infection: A Literature Review. Br. J. Community Nurs..

[35] Lin S., Li G., Huang H. (2013). Comparative Study of the Disinfection Effects of Three Types of Conjunctiva Sac Irrigations. Eye Sci..

[36] Zhou P., Li L.-F., Zhang K., Wang B., Tang L., Li M., Wang T., Sun Y., Li S., Qiu H.-J. (2022). Deletion of the H240R Gene of African Swine Fever Virus Decreases Infectious Progeny Virus Production Due to Aberrant Virion Morphogenesis and Enhances Inflammatory Cytokine Expression in Porcine Macrophages. J. Virol..

[37] Salas M.L., Andrés G. (2013). African Swine Fever Virus Morphogenesis. Virus Res..

[38] Harvey E., Holmes E.C. (2022). Diversity and Evolution of the Animal Virome. Nat. Rev. Microbiol..

[39] Rossmann M.G. (2013). Structure of Viruses: A Short History. Q. Rev. Biophys..

[40] FAO (2001). Manual on the Preparation of African Swine Fever Contingency Plans.

